# Predictive Factors of Malignancy in Cervical Paragangliomas: A Retrospective Study

**DOI:** 10.1002/hed.28250

**Published:** 2025-07-18

**Authors:** Garance Haw, Maxime Fieux, Anthime Flaus, Hélène Lasolle, Juliette Abeillon, Myriam Decaussin‐Petrucci, Dylan Pavie, Benjamin Verillaud, Philippe Herman, Patrick Feugier, Fatima Ameur, Pierre Philouze, Françoise Borson‐Chazot, Philippe Ceruse

**Affiliations:** ^1^ Hospices Civils de Lyon, Hôpital de la Croix Rousse Service d'ORL et de Chirurgie Cervico‐Faciale Lyon France; ^2^ Hospices civils de Lyon, Hôpital Lyon Sud Service d'ORL, d'Otoneurochirurgie et de Chirurgie Cervico‐Faciale France; ^3^ Université de Lyon, Université Lyon 1 Lyon France; ^4^ UMR 5305, Laboratoire de Biologie Tissulaire et d'Ingénierie Thérapeutique, Institut de Biologie et Chimie des Protéines, CNRS/Université Claude Bernard Lyon 1 Lyon France; ^5^ Hospices Civils de Lyon, Hôpital Louis Pradel Service de Médecine Nucléaire Bron France; ^6^ Centre de Recherche en Neurosciences de Lyon Inserm U1028 ‐ CNRS UMR5292 Groupement Hospitalier Est—CERMEP Bron France; ^7^ Hospices Civils de Lyon, Hôpital Louis Pradel Service d'Endocrinologie Bron France; ^8^ Hospices Civils de Lyon, Hôpital Lyon Sud Service d'Anatomopathologie France; ^9^ Hospices Civils de Lyon, Hôpital de la Croix Rousse service de radiologie Lyon France; ^10^ Assistance Publique Hôpitaux de Paris, Hôpital Lariboisière Service d'ORL et de Chirurgie Cervico‐Faciale Paris France; ^11^ Hospices Civils de Lyon, Hôpital Lyon Sud Service de Chirurgie Vasculaire et Endovasculaire LYVES France; ^12^ Hospices Civils de Lyon, Hôpital Lyon Sud Service d'Imagerie Médicale et Interventionnelle France

**Keywords:** cervical paraganglioma, imaging finding, malignancy, metastases, surgery

## Abstract

**Background:**

Early predictive factors of metastatic cervical paragangliomas (cPG) are lacking.

**Methods:**

This multicenter retrospective study included patients with at least one cPG. Metastatic cPG were defined by the histological presence of lymph node metastases or distant metastases. Clinical, radiological, intraoperative, histological, and mutational status characteristics were collected. Predictive factors of metastatic cPG were searched using logistic regression.

**Results:**

Sixty‐seven patients were included, corresponding to 86 cPG; 12.8% of these were metastatic. The seven newly identified risk factors were: presence of necrosis (OR = 12.36, 95% CI: [3.03–55.66]), extracapsular extension (OR = 33.42, 95% CI: [2.48–4752.00]), and pathological lymph nodes (OR = 25.00, 95% CI: [3.96–276.07]) on morphological imaging (MRI and/or CT); heterogeneous tumor uptake (OR = 15.5, 95% CI: [2.31–143.68]) and abnormal lymph node uptake (OR = 16.5, 95% CI: [2.04–174.94]) on functional imaging (FDG–PET–CT); invasion of adjacent tissues (OR = 34.63, 95% CI: [3.82–4602.65]) and sacrifice of noble structures (OR = 75.9, 95% CI: [7.99–10230.73]) in patients who underwent surgery.

**Conclusion:**

These risk factors could be combined to promptly identify aggressive cPG and adapt therapeutic strategy.

## 1 Introduction

Cervical paragangliomas (cPG) are rare tumors that originate from parasympathetic chromaffin tissue but rarely secrete catecholamines [1, 2]. In 40% of the cases, there is a genetic predisposition syndrome, which increases the risk of multiple localizations. Since 2017, paraganglioma (PG) has been classified by the World Health Organization (WHO) as tumors with metastatic potential [3]. Metastatic PG has been reported to have a 37% mortality rate at 5 years [4] and their diagnosis is currently made when lymph node or visceral metastases are discovered. PG are usually diagnosed between the ages of 20 and 40, except for hereditary forms that are often diagnosed earlier [5]. Metastases, however, may appear several years after the initial PG diagnosis [6]. Although surgery is currently the gold standard treatment, it is associated with high morbidity; cranial nerve palsy is reported in 11.8%–20.5% of the cases [7, 8]. Therefore, some patients are only monitored or benefit from radiotherapy for slowly progressing tumors [9], which may fail to recognize metastatic tumors, ultimately threatening vital prognosis due to both their local and distant development. The difficulty, therefore, lies in the early identification of PG, including cPG, that will become metastatic and criteria other than the metastasis itself are needed to optimize their management.

Few data are available regarding risk factors for metastases in cPG, and study results are sometimes discordant. For example, the significance of tumor size as a predictor of malignancy remains inconclusive in the literature [10, 11, 12]. Some studies show a wide variability in tumor size for metastatic and nonmetastatic PG, with small tumors (< 2 cm) also being associated with synchronous metastases [12]. Moreover, PG are often associated with pheochromocytomas, thus preventing clear conclusions from being drawn [13, 14, 15, 16]. Histological features of the tumor that appear to be associated with metastases are a high mitotic index, a capsular invasion, and necrosis [17, 18]. Nevertheless, the only criterion currently recognized as a risk factor for metastasis development is the presence of a Succinate Dehydrogenase Complex Iron–Sulfur Subunit B (*SDHB*) mutation [19, 20]. Interestingly, in our clinical practice, we observed that, during surgery, certain cPG are poorly limited and present with a macroscopic invasion of the adjacent nerve, while most cPG are well encapsulated: this led us to the hypothesis that such macroscopic aspects could be associated with metastatic cPG.

The main aim of this study was to identify clinical, laboratory, imaging, surgical, and histological predictive factors of metastatic cPG.

## 2 Subjects and Methods

### 2.1 Study Design and Ethics

This multicenter retrospective study was conducted on patients included between 2009 and 2022 from two tertiary referral centers in France. This study included human participants and was conducted in accordance with the Declaration of Helsinki. It was approved by the institutional ethics committee (*Comité Scientifique et Ethique des Hospices Civils de Lyon*, no. 23‐5015). All data were anonymized. Written consent was not required given the retrospective design of the study. However, written informed consent was obtained before any genetic testing for the patients concerned. Inclusion criteria were: the presence of at least one cPG confirmed on morphological imaging (at least one computed tomography [CT] scan or magnetic resonance imaging [MRI] available), an ear–nose and throat (ENT) examination with an evaluation of the cranial nerves, and a laboratory search for *SDH* germline mutation. All included patients had at least a 1‐year follow‐up before inclusion. Locations other than cervical, such as tympano‐jugular PG, were excluded.

### 2.2 Variables Collected

Data were collected from electronic medical records, at diagnosis and during follow‐up: age at diagnosis, sex, mutational status (on blood sample), symptomatology, tumor size and volume, location, multifocality, functional status, therapeutic management (surgery, radiotherapy, monitoring), findings during surgery (contiguous invasion, sacrifice of noble elements such as nerves or major arteries), local recurrence, existence or appearance of regional or distant metastases, and histological results in case of surgery. In case of cPG of the vagus, the sacrifice of the X would be considered as sacrifice of a noble element. In contrast, sacrificing the carotid artery in case of cPG of the carotid artery was not considered as sacrifice of a noble element. Multifocality was defined by the presence of at least two PGs and/or pheochromocytoma regardless of their location. The presence of metastases was not a criterion for multifocality.

According to our clinical practice, each case is discussed during multidisciplinary tumor board meetings to determine the appropriate therapeutic strategy. Surgery is performed only if the surgeon considers that a complete tumor resection can be achieved; otherwise, an alternative treatment is proposed. Consequently, no subtotal resection is performed. When necessary, wide resections involving neural and/or vascular sacrifice are carried out. A minimum selective neck dissection of Levels IIa and III was performed in all patients to ensure vascular axis exposure. The procedure was extended to include additional nodal levels in cases where intraoperative assessment revealed lymph nodes with pathological characteristics. Two characteristics were collected from the operative report: invasion of surrounding structures by the tumor and the necessity to sacrifice elements adjacent to the tumor (nerves X, XI, XII, internal jugular vein), referred to in this paper as “noble elements.” For patients who underwent surgery, histological data were collected from the postoperative report and interpreted by a pathologist from the reference center.

All the morphological imaging (CT and MRI) performed at the time of the initial diagnosis were reviewed by a team of ENT physicians and a radiologist. CT scans consisted of a spontaneous contrast acquisition followed by an acquisition after injection of iodinated contrast media at arterial time. The MRI protocol consisted of acquisitions in T1, T2, and 3DTOF sequences, which were completed by angio‐MRI sequences after injection of gadolinium. This protocol tends to confirm the diagnosis when showing a typical T1 hyposignal, heterogeneous T2 hypersignal with areas of flow voids inducing a salt and pepper look, and intense enhancement after injection. The imaging features of particular interest were the presence of intra‐tumoral necrosis, extracapsular extension, and the presence of adenopathy. A lymph node was defined as pathological if its smaller axial diameter exceeded 10 mm. As cPGs are well encapsulated tumors, the diagnosis of extracapsular extension was based on the observation of a break in the continuity of the tumor capsule, possibly leading to invasion of adjacent structures. This observation was preferentially made on T1 sequences. Tumor size was determined by the largest transverse diameter on the initial CT or MRI scan. Tumor volume was determined on morphological imaging (CT or MRI) by measuring the three largest orthogonal diameters: the maximum height *d*_1_ was measured in the coronal plane while the anteroposterior diameter *d*_2_ and the lateral diameter *d*_3_ were measured in the axial plane. The volume was then calculated on the basis of an ellipsoid: $V=d_{1}\times d_{2}\times d_{3}\times \left(\pi /6\right)$. For patients who also underwent a functional imaging (Fluorodeoxyglucose positron electron tomography [FDG PET] CT) at diagnosis, the scans were reviewed by a nuclear physician who was only informed of the diagnosis and blinded to the information from the medical records. SUVmax was obtained from the Syngo.Via interpretation software (Siemens Healthcare GmbH, Erlangen, Germany), which selected the voxel with the maximum uptake inside the region of interest (ROI) and to which the following formula was applied: $\frac{\text{Activity concentration}\;\left(\mathrm{kBq}/\mathrm{mL}\right)}{\text{injected dose}\;\mathrm{at}\;\text{the time of examination}\left(\mathrm{kBq}\right)/{\text{patient}}^{\prime }s\;\text{weight}\;\left(g\right)}$.

The diagnosis of malignancy was confirmed by the histological presence of lymph node metastasis or distant metastasis, that is, the presence of chromaffin tissue in an organ where it is not usually found (bone, liver, lung, etc.); this allowed us to define two groups: metastatic cPG and nonmetastatic cPG. The diagnosis of local recurrence was based on the reappearance of tumor tissue at the initial site during follow‐up, with histological evidence. The following germline mutations were systematically tested at diagnosis on a blood sample using next‐generation sequencing (NGS) target enrichment: *SDHA*, *SDHB*, *SDHD*, *VHL*. The presence of a mutation in a first sample was confirmed in a second sample by Sanger or multiplex ligation‐dependent probe amplification sequencing.

### 2.3 Statistical Analysis

Discrete variables are summarized as frequencies and percentages and continuous variables as means with standard deviations (SDs). At diagnosis, comparisons between the metastatic cPG and cPG groups were carried out using the chi‐square test for categorical variables and the independent two‐sample *t*‐test for continuous variables (in case of noncompliance with the underlying hypotheses, Fisher's exact test and Wilcoxon test were used). An odds ratio (OR) coefficient with its 95% confidence interval (CI) was calculated for each characteristic included in a univariable logistic regression model to identify predictive factors of metastasis. Two logistic regressions were carried out: one was carried out on the total population to search for clinical, laboratory, and imaging factors and one on the population having undergone surgery to search for surgical and histological factors. Variables were included in the univariate analysis if they were known to be associated with metastatic PG or if the authors thought they could be of interest. A variable with an OR > 1 was considered as a risk factor for metastatic cPG while a variable with an OR < 1 was considered as a protective factor. No multivariable analysis could be performed because of the small sample size of the metastatic cPG group. Contingency tables were built based on cPG metastasis and tumor size. The corresponding receiver operating characteristic (ROC) curve was plotted and the threshold with the highest Youden index (combined sensitivity and specificity) was selected [21]. Results were considered statistically significant for a *p* ≤ 0.05. Statistical analyses were performed with R version 4.3.3 (R Foundation for Statistical Computing, Vienna, Austria).

## 3 Results

### 3.1 Description of the Population

A total of 67 patients with cPG were included. Their mean (SD) age was 53 (14.63) years, and 50.7% were women. The mean (range) follow‐up duration was 65 (12–159) months. Among all patients, 67.2% (45/67) had multifocal disease (cervical and/or distant), corresponding to 86 cPG. Metastatic cPG accounted for 12.8% of the tumors (11/86), 45.5% (5/11) of which were identified at diagnosis and 54.5% (6/11) at follow‐up. Metastatic cPG and multifocal disease occurred in four patients, in whom metastases were attributable to only one cPG: two were diagnosed as metastatic based on the presence of ipsilateral lymph node metastases, while the other two developed metastases before developing other localizations. Overall, 11 of 67 patients (16.4%) had metastatic cPG. Isolated lymph node metastases were found in 27.3% (3/11) of these patients. Among the remaining 8 of 11 patients with distant metastases, one had metastases at diagnosis, three were diagnosed within 2 years, one was diagnosed at 5 years, and three were diagnosed between 7 and 8 years.

All 67 patients had genetic testing: one had a VHL mutation, two had SDHC mutations, four had SDHA mutations, nine had SDHB mutations, and 24 had SDHD mutations.

Of the 86 cPG, 73% (63/86) were carotid body cPG and 27% (23/86) were vagal cPG. Overall, 55.8% (48/86) of the tumors were treated surgically, nine of which were irradiated postoperatively. Adjuvant radiotherapy was indicated in cases of incomplete resection on histology or intraoperative evidence of aggressive tumor behavior. Exclusive radiotherapy was proposed for 13.9% (12/86) of the cPG, and 30.2% (26/86) of the tumors benefited from a simple monitoring. The clinical characteristics of the patients according to the treatment chosen are described in Table 1.

**TABLE 1:** Clinical characteristics of the cervical paragangliomas according to the type of treatment received (*n* = 86).

	Surgery (*n* = 48)	Radiotherapy (*n* = 11)	Monitoring (*n* = 27)	*p*
Age (years), median (range)	42 (20–75)	45 (23–71)	57 (37–87)	0.001
Sex, *n* (%)				0.609
Female	27 (56.2)	6 (54.5)	12 (44.4)	
Male	21 (43.8)	5 (45.5)	15 (55.6)	
Localization, *n* (%)				0.002
Carotid body	40 (83.3)	4 (36.4)	19 (70.4)	
Vagal	8 (16.6)	7 (63.6)	8 (29.6)	
Size (mm), median (range)	32 (8–77)	40 (14–65)	22 (6–67)	0.032
Malignant, *n* (%)	8 (16.7)	3 (27.3)	0 (0)	0.018
SDHA, *n* (%)	2 (4.5)	0 (0)	3 (11.1)	0.406
SDHB, *n* (%)	6 (13.6)	3 (30)	0 (0)	0.023
SDHD, *n* (%)	23 (52.3)	5 (50)	11 (40.7)	0.665
Duration of follow‐up (months), median (range)	59 (12–130)	97 (15–140)	54 (17–150)	0.669

### 3.2 Identification of Clinical, Laboratory, and Imaging Predictive Factors

**FIGURE 1:**
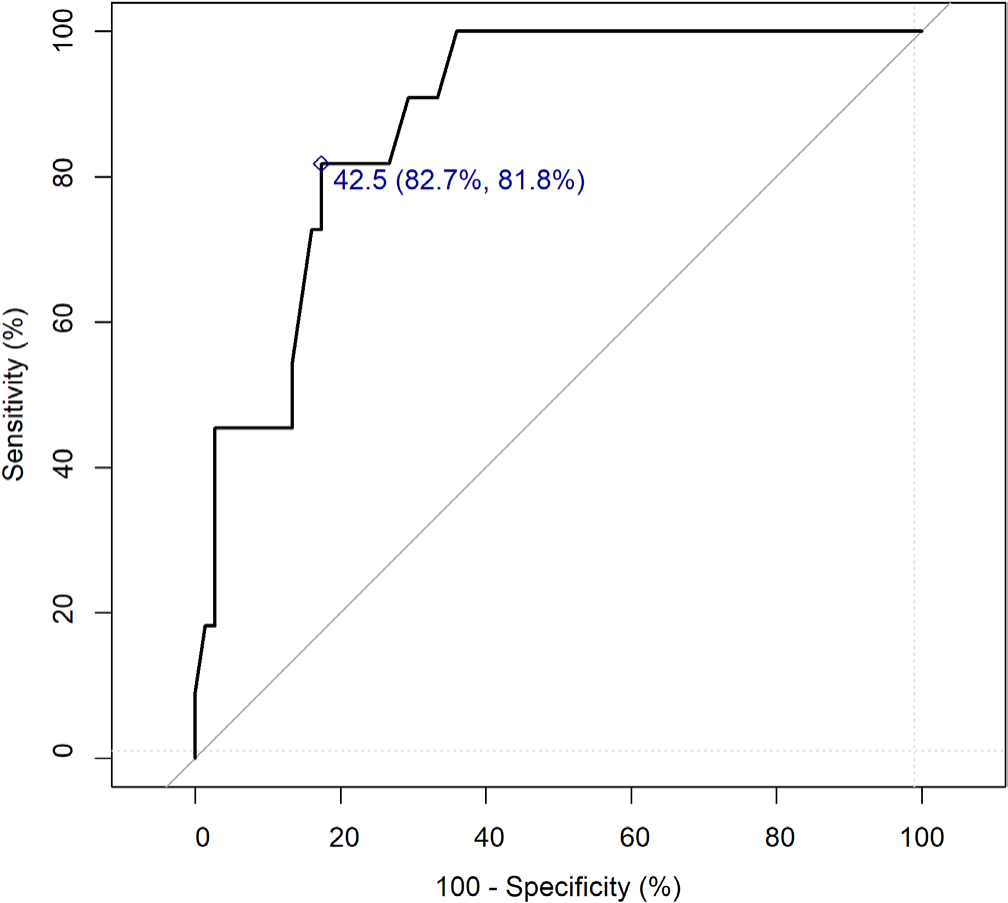
ROC curve used to determine the tumor size threshold to distinguish between nonmetastatic and metastatic cervical paragangliomas (cPG). The ROC curve to identify metastatic cPG based on tumor size is given with the highest Youden index in mm, and the corresponding sensitivity and specificity in percentage. [Color figure can be viewed at wileyonlinelibrary.com]

After univariate analysis on the total population of cPGs (*n* = 86), nine risk factors of metastasis development and one protective factor were identified. The presence of the *SDHB* mutation (OR = 7.43, 95% CI: [1.55–35.31], *p* = 0.01) was identified as a predictive factor while the presence of an *SDHD* mutation was a protective factor (OR = 0.2, 95% CI: [0.03–0.84], *p* = 0.048). On either CT or MRI, tumor size (OR = 1.11, 95% CI: [1.06–1.19], *p* < 0.001) and tumor volume (OR = 1.08, 95% CI: [1.04–1.14], *p* < 0.001) were significantly associated with an increased risk of metastatic cPG. On both MRI and CT imaging, the presence of intra‐tumoral necrosis (OR = 12.36, 95% CI: [3.03–55.66], *p* = 0.0005 and OR = 12.91, 95% CI: [2.00–99.63], *p* = 0.007, respectively), pathological node(s) (OR = 25.00, 95% CI: [3.96–276.07], *p* = 0.0006 and OR = 65.00, 95% CI: [4.27–9709.75], *p* = 0.002, respectively), and extracapsular extension (OR = 33.42, 95% CI: [2.48–4752.00], *p* = 0.008 and OR = 30.33, 95% CI: [1.41–4712.16], *p* = 0.030, respectively) were significantly associated with an excess risk of metastatic cPG. On functional imaging (FDG PET/CT), heterogeneous tumor fixation (OR = 15.5, 95% CI: [2.31–143.68], *p* = 0.007), fixation of one or more locoregional lymph nodes (OR = 16.5, 95% CI: [2.04–174.94], *p* = 0.01), and the $\frac{\text{SUVmax tumor}}{\text{SUVmax liver}}$ ratio (OR = 1.34, 95% CI: [1.097–1.757], *p* = 0.023) were predictive of metastatic cPG (Table 2). Based on the ROC curve analysis, the tumor size threshold offering the best sensitivity and specificity for distinguishing between nonmetastatic and metastatic cPG was 4.25 cm (Se = 82.7%; Sp = 81.8%; Figure 1).

**TABLE 2:** Imaging risk factors of metastatic cervical paragangliomas determined by logistic regression in the total population (*n* = 86).

	OR	95% CI	*p*
Morphological imaging (either CT or MRI)			
Tumor size	1.11	1.06–1.19	< 0.001*
Tumor volume	1.08	1.04–1.14	< 0.001*
Localization			
*Carotid body*	—	—	—
Vagal	1.41	0.28–5.71	0.643
MRI			
Necrosis			
*Absent*	—	—	—
Present	12.36	3.03–55.66	0.0005*
Pathological node			
*Absent*	—	—	—
Present	25.00	3.96–276.07	0.0006*
Extracapsular extension			
*Absent*	—	—	—
Present	33.42	2.48–4752.00	< 0.008*
CT scans			
Necrosis			
*Absent*	—	—	—
Present	12.91	2.00–99.63	0.007*
Pathological node			
*Absent*	—	—	—
Present	65	4.27–9709.75	0.002*
Extracapsular extension			
*Absent*	—	—	—
Present	30.33	1.41–4712.16	0.03*
Functional imaging			
SUVmax	1.08	1–1.17	0.054
Fixation			
*Homogeneous*	—	—	—
Heterogeneous	15.5	2.31–143.68	0.007*
Nodal fixation			
*No*	—	—	—
Yes	16.5	2.04–174.94	0.01*
Ratio SUVmaxtumor/SUVmaxliver	1.34	1.097–1.757	0.023*

*Note*: The results provided are odds ratios (OR) in relation to the category chosen as reference for the categorical variables (categories italicized in the table). If OR > 1, the variable is a risk factor for metastatic paraganglioma; conversely, if OR < 1, the variable is a protective factor.

* *p* < 0.05.

### 3.3 Identification of Surgical and Histological Predictive Factors

After univariate analysis on the subgroup of cPG that underwent surgery (*n* = 48), 3 risk factors of metastasis were identified: invasion of adjacent tissues (OR = 34.63, 95% CI: [3.82–4602.65], *p* < 0.0001), sacrifice of noble elements during the surgery (OR = 75.9, 95% CI: [7.99–10230.73], *p* < 0.0001), and necrosis on histological findings (OR = 26.82, 95% CI: [1.86–3907.26], *p* = 0.0145; Table 3).

**TABLE 3:** Risk factors of metastatic cervical paragangliomas determined by logistic regression in the subgroup of patients who underwent surgery (*n* = 48).

	OR	95% CI	*p*
Clinical data			
Sex			
*Female*	—	—	—
Male	3.39	0.90–16.42	0.088
Surgical data			
Invasion of adjacent tissues			
*No*	—	—	—
Yes	34.63	3.82–4602.65	< 0.0001*
Sacrifice of noble elements			
No	—	—	—
Yes	75.9	7.99–10230.73	< 0.0001*
Histological data			
Necrosis			
*Absent*	—	—	—
Present	26.82	1.86–3907.26	0.0145*
Ki67	1.10	0.93–1.30	0.232
Loss of expression of *SDHB*			
*No*	—	—	—
Yes	1.5	0.18–14.78	0.707
Size	1.08	1.017–1.17	0.023*
Mitotic figures	1.15	0.599–1.93	0.625

*Note*: The results provided are odds ratios (OR) in relation to the category chosen as reference for the categorical variables (categories italicized in the table). If OR > 1, the variable is a risk factor for metastatic paraganglioma; conversely, if OR < 1, the variable is a protective factor.

* *p* < 0.05.

## 4 Discussion

Twelve risk factors of metastatic cPG were identified in the present study: the presence of an *SDHB* mutation, tumor size and volume on morphological imaging (either CT or MRI), the presence of intra‐tumoral necrosis, lymph node(s), and extracapsular extension on both MRI and CT, the presence of heterogeneous tumor uptake, pathological nodal uptake, and $\frac{\text{SUVmax tumor}}{\text{SUVmax liver}}$ ratio on FDG PET/CT, the sacrifice of noble elements and invasion of adjacent tissues during surgery, and necrosis on histological findings.

First, the present cohort appears representative of the cPG population, notably regarding the proportion of patients who developed metastases, which was concordant with the 3%–15% reported in the literature [22, 23]. However, only a third of the patients had lymph node involvement (including one with initial adenopathy and metachronous distant metastasis) and two thirds had distant metastases, which is often the opposite in the literature, despite variability in published results [22, 23, 24]. Moreover, more than half of the patients had an *SDH* mutation herein, which is slightly higher than the rates found in other studies (around 30%) [25, 26].

The present study did not find age to be a significant risk factor for metastatic cPG, a result in line with that of Feng et al. [27], but which differs from other series [11, 28]. The latter, however, did not take into account evident confounding factors, such as localization (i.e., cPG) and mutational status. In the present cohort, the presence of the *SDHB* mutation was found to be a risk factor of metastatic cPG, confirming the results of previous studies. Brouwers et al. found up to 40% of *SDHB* mutations in their cohort of metastatic PG (*n* = 44) [19], while Amar et al. found reduced survival in patients with the *SDHB* mutation (*n* = 54) [29]. Conversely, the *SDHD* mutation was identified herein as a protective factor. However, the several‐month delay in the obtention of germline mutation results undermines their use as prognostic factors. Thus, early predictive factors are needed to promptly adapt the therapeutic strategy.

On morphological imaging, tumor size was identified herein as a risk factor for metastasis, corroborating numerous studies on the subject [7, 10, 12]. Moreover, the threshold that offered the best performance for discriminating between metastatic and nonmetastatic cPG in the present study was very close to the 4.5 cm threshold previously reported, beyond which the risk of developing metastasis was found to be significant. In the same study, which included 106 patients, the authors also showed that the impact on survival was significant for tumors larger than 5.5 cm [10]. On CT and MRI, extracapsular extension, presence of necrosis, and pathological lymph node(s) were more frequent in the metastatic population. In 2008, Jacques et al. [15] demonstrated a correlation between heterogeneity on MRI and the presence of necrosis on pathology in 44 pheochromocytomas and that malignant tumors appeared more often heterogeneous on MRI. No further studies have been carried out on this subject since. On functional imaging, heterogeneous tumor metabolism and metabolic lymph node involvement on FDG PET/CT were both significantly associated with metastatic progression in the total population. Heterogeneous uptake on PET may be explained by the presence of intra‐tumoral necrosis that does not retain the tracer. Metastatic tumors also had a higher $\frac{\text{SUVmax tumor}}{\text{SUVmax liver}}$ ratio. In their population of 23 recurrent PGs, Fikri et al. [16] found a difference in SUVmax distribution between metastatic and localized tumors and reported that metastatic progression was correlated with an SUVmax greater than 9.2. SUVmax reflects altered tumor glycolytic metabolism and therefore tumor aggressiveness [30, 31]. This is supported by several studies, which have demonstrated that, in various malignant tumors, high SUV values are associated with more aggressive diseases [32, 33]. The use of the ratio seemed more reliable than the study of SUVmax alone, as it allows to overcome the variability in acquisition conditions, and thus improves the comparability of tumor fixation. Another interesting hypothesis is that aggressive tumors, through dedifferentiation and loss of somatostatin receptor (SSTR) expression, may be less receptive to specific tracers such as 68‐Ga‐DOTATOC, a radiolabeled somatostatin analog with high affinity for SSTR [34]. This hypothesis could not be investigated herein, as only a small number of patients had undergone DOTATOC PET, but this hypothesis deserves to be studied in a dedicated population.

Regarding surgical data, the invasion of adjacent tissues and the sacrifice of noble elements were identified as risk factors. Similarly, in the study by Zhang et al., metastatic cPG were more often symptomatic preoperatively and more frequently required the sacrifice of noble elements (intraoperative reconstruction or suturing of vascular wounds) [7]. These elements suggest that metastatic tumors are more aggressive locally and are therefore subject to a greater risk of intraoperative complications. Identifying these surgical criteria would be an additional argument for considering adjuvant radiotherapy [35], since the latter has been shown to be associated with better survival than surgery alone for aggressive tumors [36].

Histologically, only the presence of necrosis appeared to be a risk factor for metastasis in the subgroup of patients who underwent surgery. Other histological risk factors, such as Ki67, extracapsular extension, and mitotic count described in numerous studies [37, 38, 39], were not found herein. However, these factors are often combined into multivariable scores [17, 18] since their predictive value, when considered separately, is of little clinical relevance. For example, the Ki67 proliferation index has been shown to be specific but not very sensitive [40]. Unfortunately, the histological prognostic scores developed by Thompson et al. (PASS [18]) and Kimura et al. (GAPP [17]) could not be used herein due to a lack of certain histological data required for calculation. In addition, PASS was only developed for pheochromocytomas. Finally, due to the retrospective design of the present study, numerous histological data were missing, which may explain the lack of significant results.

Overall, these results suggest the existence of a variable spectrum of cPG aggressiveness, which can be predicted based on the presence of at least one of the risk factors identified herein. This idea corroborates the results by Harley et al. who investigated the prognostic value of tumor aggressiveness in cPG [36]. Local aggressiveness was determined by the American ICD‐O‐3 classification, which classifies tumors into three categories according to histological, cytological, and imaging findings (benign, invasive, and uncertain or borderline) and the authors showed that tumor aggressiveness was significantly associated with survival. Based on these findings, we propose to consider a new category of cPG, called aggressive cPG (APG). The latter would be defined by either the presence of at least one imaging and one surgical risk factor for surgically treated tumors or the presence of at least two imaging risk factors for non‐operated tumors. APG should benefit from close monitoring and long‐term follow‐up, as metastases can develop up to several years after the diagnosis [41, 42]. Promptly identifying APG in daily routine would also enable physicians to opt for surgical management, as it has been shown to have a significant impact on survival [6, 43]. In these tumors, surgery of the primary site should be accompanied by surgery of the lymph node areas to avoid missing lymph node metastases [22, 44, 45]. Nevertheless, the morbidity of surgery must be taken into account in the benefit–risk balance, particularly in the case of vagal tumors, for which the risk of sequelae is greater [46].

The main limitation of the present study lies in its retrospective design, which led to a large number of missing data. Another limitation is the small size of the population, which can be attributed to the rarity of cPG. Second, a potential selection bias, due to the inclusion of patients only from tertiary referral centers, may have overestimated the proportion of metastatic cPG. Finally, the sacrifice of the X in case of cPG of the vagus was almost systematic during surgery and thus considered a sacrifice of noble element. While this may represent a potential source of bias, its impact remains limited due to the very small number of surgically treated vagal cPG in this cohort (*n* = 8) compared to carotid cPG (*n* = 40). Nevertheless, the present study enabled the identification of 12 risk factors of metastatic cPG, which could be combined to promptly identify APG and adapt therapeutic strategy.

## 5 Conclusion

In a cohort of 86 cPG, we identified new risk factors of metastatic progression, such as the presence of extracapsular extension, necrosis, and pathological lymph nodes on MRI and CT scan as well as the presence of heterogeneous tumor fixation and lymph node fixation on functional imaging (FDG–PET). During surgery, the metastatic tumors more often invaded adjacent structures, requiring the sacrifice of noble elements. Knowledge of these criteria could enable better adaptation of therapeutic management and reduce the occurrence of metastasis.

## Author Contributions

All authors contributed to the study conception and design as well as the drafting of the article. M.F. was responsible for the statistical analysis of the data. All authors approved the final version of the manuscript.

## Ethics Statement

This study included human participants and was conducted in accordance with the Declaration of Helsinki. It was approved by the institutional ethics committee (Comité Scientifique et Ethique des Hospices Civils de Lyon, no. 23‐5015). All data were anonymized.

## Consent

Written consent was not required given the retrospective design of the study. However, written informed consent was obtained before any genetic testing for the patients concerned.

## Conflicts of Interest

The authors declare no conflicts of interest.

## Data Availability

The data that support the findings of this study are available on request from the corresponding author. The data are not publicly available due to privacy or ethical restrictions.
